# Editorial Note: School-Located Influenza Vaccination Reduces Community Risk for Influenza and Influenza-Like Illness Emergency Care Visits

**DOI:** 10.1371/journal.pone.0291903

**Published:** 2023-09-19

**Authors:** The PLOS ONE Editors

Following the publication of this article [1], the corresponding author informed *PLOS ONE* that they became aware of an issue with the collection method that may have impacted the data from the ESSENCE surveillance system reported in this study.

Specifically, they reported that part way through the study, one of the two major acute care hospitals in Alachua County switched from recording the chief complaint using a free text box to using a dropdown menu. It was not possible to determine if this change impacted the counting of influenza or influenza-like illness in the data from this hospital.

To determine if the results of this study are supported in light of this issue, the corresponding author directed the editors to alternative analysis of the key results using data from the state Agency for Health Care Administration based on discharge diagnoses rather than chief complaints. This analysis was published in a PhD thesis [2] (sections are described in S1 File with this notice).

An independent expert reviewed the analyses in the thesis [2] and the original article [1]. They concluded that the methods and results in the thesis are appropriate and valid, and that these results support the conclusions of the article [1]. The corresponding author acknowledges the reviewer’s comments, which can be found in S1 File.

The *PLOS ONE* Editors issue this Editorial Note to notify readers of the issue that may have affected the original dataset and to direct readers to the supplementary analysis which has been found to support the conclusions of this article.

## Supporting information

S1 File Reviewer’s comments. (DOCX)
