# Supplementary material for: Editorial Note: School-Located Influenza Vaccination Reduces Community Risk for Influenza and Influenza-Like Illness Emergency Care Visits
Source: PLoS One. 2023 Sep 19;18(9):e0291903. doi: 10.1371/journal.pone.0291903 (PMC10508592; doi:10.1371/journal.pone.0291903)
Supplement: S1 File — (DOCX) [file pone.0291903.s001.docx]

**School-Located Influenza Vaccination Reduces Community Risk for Influenza and Influenza-Like Illness Emergency Care Visits**

<https://doi.org/10.1371/journal.pone.0114479>

**Alternative analysis in PhD thesis**

Results of alternative analysis using data from the state Agency for Health Care Administration based on discharge diagnoses were included in the following parts of a PhD thesis in epidemiology at the University of Washington (<https://digital.lib.washington.edu/researchworks/handle/1773/38077>).

- Page 4-6, Section 1.1.2: Discussion of issues identified in Tran et al (2014), coding changes in ESSENCE system chief complaints, explanation of alternative data from the Florida Agency for Hospital Care Administration (AHCA).
- Pages 91-94, Sections 4.7.1 and 4.7.2: analysis of effectiveness of school-location influenza vaccination programme in Alachua Region using ACHA data.

**Editor queries to reviewer:**

I would be very grateful if you would please provide your thoughts in response to the following queries:

1. Is the methodology described in the article and the re-analysis in the PhD thesis appropriate and valid to support the conclusions of the study?
2. Is the methodology described in sufficient detail to allow for reproducibility, and if not, what further information would be required to ensure reproducibility?
3. Are the limitations of the study design and their impact on the strength of the conclusions adequately acknowledged in the article?
4. In the introduction, is the wider literature that was available at the time (2014) adequately and accurately represented?
5. Do you have any other comments on this article, in particular regarding whether any additional information would be required to ensure it reports a reproducible study with adequately supported conclusions.

**Reviewer comments:**

1) From my reading, the methods and analyses in Fisher’s thesis are appropriate and valid and do support the conclusions of the study.
2) The methods are adequately reported and appear to be reproducible.
3) The study limitations are overall sufficiently considered. However, a few comments: I would have preferred to see the analysis outcomes reported as specified in the protocol, within the Abstract and Methods, and the results for these presented accordingly (bearing in mind biases in outcome reporting, etc). Secondly, statements about shedding virus should specify how the virus was identified – via identification of the presence of viral RNA alone gives very little information, whereas identification of replication-competent virus (via cell culture of biological samples), or even RNA identification coupled with information on viral load and changes over time have far greater relevance to understanding viral transmission.

I would prefer more emphasis and consideration of the results that show indirect effectiveness for the over 65 age group showed no impact. It may be that vaccinating only the very youngest would bring the greatest benefit. To establish a vaccination program intended at least in part to benefit others than those receiving the vaccine requires considerations of the ethics, and if an expected benefit to the other age groups is not forthcoming, that may influence decisions on the program.

Finally, any study of any intervention should consider both benefits and harms. Although this was a study using published rates of disease conditions, harms should be mentioned and whether any data could be reviewed to assess these.

4) This is a difficult question to answer. The background to a study sets out its rationale and ideally marshalls facts from which to set out a hypothesis. I personally would prefer to set the issue of vaccinations within a wider context – i.e. reduction of rates per se may not in fact be ultimately desirable (are there long term sequalae, impacts on other conditions, etc etc). However, I would not generally expect a paper’s introduction section to include this wider consideration.
5) Aside from the above comments, my reading is that the study provides a reasonable view of the findings in the light of possible and probable biases.
